# Global COVID-19 vaccine acceptance rate: Systematic review and meta-analysis

**DOI:** 10.3389/fpubh.2022.1044193

**Published:** 2022-12-08

**Authors:** Dechasa Adare Mengistu, Yohannes Mulugeta Demmu, Yohanis Alemeshet Asefa

**Affiliations:** Department of Environmental Health, College of Health and Medical Science, Haramaya University, Harar, Ethiopia

**Keywords:** vaccine acceptance, vaccine hesitancy, COVID-19, coronavirus, 2019, SARS-CoV-2, vaccine rejection, global

## Abstract

**Background:**

A vaccine against COVID-19 is a vital tool in managing the current pandemic. It is becoming evident that an effective vaccine would be required to control COVID-19. Effective use of vaccines is very important in controlling pandemics and paving the way for an acceptable exit strategy. Therefore, this systematic review and meta-analysis aims to determine the global COVID-19 acceptance rate that is necessary for better management of COVID-19 pandemic.

**Methods:**

This review was conducted based on Preferred Reporting Items for Systematic Reviews and Meta-Analysis protocols and considered the studies conducted on acceptance and/or hesitancy of COVID-19 vaccine. Articles were searched using electronic databases including PubMed, Scopus, Web of Science, Embase, CINAHL, and Google Scholar. The quality of the study was assessed using the Joanna Briggs Institute (JBI) critical assessment tool to determine the relevance of each included article to the study.

**Results:**

Of the 6,021 articles identified through the electronic database search, 68 articles were included in the systematic review and meta-analysis. The global pooled acceptance rate of the COVID-19 vaccine was found to be 64.9% [95% CI of 60.5 to 69.0%]. Based on the subgroup analysis of COVID-19 vaccine acceptance rate by the World Health Organization's region, the countries where the study was conducted, occupation, and survey period, the prevalence of COVID-19 vaccine acceptance rate was 60.8% [95% CI: 56.3, 65.2%], 61.9% [95% CI: 61.3, 62.4%], 81.6% [95% CI: 79.7, 83, 2%] and 64.5% [95% CI: 60.3, 68.5%], respectively.

**Conclusions:**

This review revealed the variation in the level of COVID-19 vaccine acceptance rate across the world. The study found that the overall prevalence of COVID-19 vaccine acceptance was 64.9%. This finding indicated that even if the COVID-19 vaccine is developed, the issue of accepting or taking the developed vaccine and managing the pandemic may be difficult.

## Introduction

Corona virus disease 2019 (COVID-19) has spread drastically throughout the world, since the first case of COVID-19 disease was reported in Wuhan, China (1), and has rapidly become a major public health concern (2). Vaccination has played a fundamental role in global public health, leading to increased life expectancy (3) and is one of the most cost-effective ways of avoiding the disease and currently prevents between two and three million deaths per year (4). It is becoming evident that an effective vaccine would be required to control COVID-19 (7). Effective use of vaccines is necessary to reduce the social and economic burden and to prepare the way for an acceptable exit strategy from the COVID-19 pandemic (8). Vaccination hesitancy and anti-vaccination movements are increasing and need critical attention (9–11). Similarly, a vaccine against COVID-19 is a vital tool in managing COVID-19 pandemic (5, 6).

Currently, vaccination rates have fallen and public confidence in vaccines has been inconsistent (6, 13) and various studies have reported a declining level of willingness to accept the COVID-19 vaccine (14). Globally, the intention of being vaccinated against the COVID-19 pandemic is declining from time to time (8). According to the World Health Organization (WHO), vaccine hesitancy has become an emerging global issue and has been identified as one of the top ten threats to global health in 2019 (12).

Although vaccines are developed against COVID-19, many factors compromise the acceptance of the vaccine against COVID-19 and become a public concern (13, 15). Furthermore, transparent and effective communication efforts are essential to reduce misinformation and vaccine hesitancy and build trust to ensure adequate vaccination coverage will be achieved (8).

Previously, several studies have been conducted and many literatures have been published to capture and address many issues regarding the COVID-19 pandemic. However, to the level of our knowledge, there is no adequate studies that have been investigated that provide the global pooled acceptance or hesitancy of the COVID-19 vaccine. Therefore, this systematic review and meta-analysis was aimed to determine the acceptance rate of the COVID-19 vaccine across the world, which is necessary to understand the acceptance or hesitancy of the vaccine in different contexts and can be an input for others pandemics.

## Materials and methods

This systematic review and meta-analysis was conducted under the Preferred Reporting Items for Systematic Reviews and Meta-Analysis (PRISMA) guidelines (16).

### Eligibility criteria

Studies that met the following inclusion criteria were included in the systematic review and meta-analysis. The inclusion criteria considered in this review include:-

- Study population: All populations regardless of their age, occupation, ethnicity, gender, etc.
- Outcomes: The articles aimed to determine COVID-19 vaccine hesitancy and/or acceptance that provided a quantitative outcome were included in the study.
- Language: Articles written in English.
- Types of articles: Peer-reviewed full text, original, and published articles.
- Publication year: Studies published since the emergency of COVID-19 to the study period (March 2020 to June 2022).
- Study regions / locations: Not specified (not limited).

However, articles not freely available, not peer-reviewed articles or preprints, editorial papers, reports, short communications, review articles, the article did not provide an outcome of interest and high risk of bias articles were excluded from this study.

### Information sources and search strategy

Article searches were performed using main key terms or keywords such as COVID-19, vaccine hesitancy, vaccine acceptance and intention to take vaccine, and Medical Subject Headings (MeSH) in combination with Boolean logic operators (“AND,” “OR,” and “NOT”). The articles were searched from PubMed, Scopus, Web of Science, Embase, CINAHL, and Google Scholar. References within eligible articles were further screened for additional articles. The articles were searched from February 01 to March 29, 2021 and May 02 to June 26, 2022 on PubMed, Scopus, Embase, and Google Scholars, while the search on Web of Science, CINAHL, and Google was made from 15 February to 31 March 2021. Articles published from March 2021 to June 2022 were searched from the included electronic databases according to their own searching strategies (Supplementary File I).

### Study selection

The study selection process was performed using the PRISMA flow chart, indicating the number of articles included in the systematic review and articles excluded from the study with the reasons of exclusion. Following the search for articles through the included electronic databases, duplicate articles were removed using the ENDNOTE software version X5 (Thomson Reuters, USA). After duplicated articles were removed, the authors (DM, YA, and YD) independently screened the articles based on their titles and abstracts by applying the inclusion criteria.

Furthermore, the full text of the relevant articles was further read in detail and the inclusion criteria independently evaluated by the authors (DM, YA, and YD). Any disagreements made with respect to the inclusion of studies were resolved by consensus after discussion. Finally, studies that met the criteria were included in the systematic review and meta-analysis.

### Data extraction

The data were extracted by the authors (DM, YA, and YD) independently. Predetermined tabular format consisting of study characteristics including publication year, survey period, country where the study was conducted, number of respondents, and outcome (COVID-19 vaccine acceptance/hesitancy rate) using Microsoft Excel, 2016 (Supplementary File II). Any disagreement made between the authors was resolved through discussion after the same procedures were repeated.

### Data quality assessment

The selected articles were subjected to a rigorous independent assessment using a standardized critical assessment tool, Joanna Briggs Institute (JBI) Critical Assessment Tools for prevalence studies (17). The evaluation tools have the following nine evaluation criteria/ parameters; (1) appropriate sampling frame; (2) proper sampling technique; (3) adequate sample size; (4) description of the study subject and setting description; (5) sufficient data analysis; (6) use of valid methods for identifying conditions; (7) valid measurement for all participants; (8) use of appropriate statistical analysis and (9) adequate response rate.

The authors (DM, YA, and YD) assessed the quality of the included studies. Based on the items in the above appraisal tool, the articles were classified as high quality (80% and above), moderate (60–80% score), and low quality (<60% score). Articles with a score ≥60% (articles has high and moderate quality) were included in the review, while those with low quality were excluded from the study. Finally, the disagreements made among the authors (DM, YA, and YD) were resolved by discussion and repeating the same procedures.

### Outcome measures

**Figure 1:**
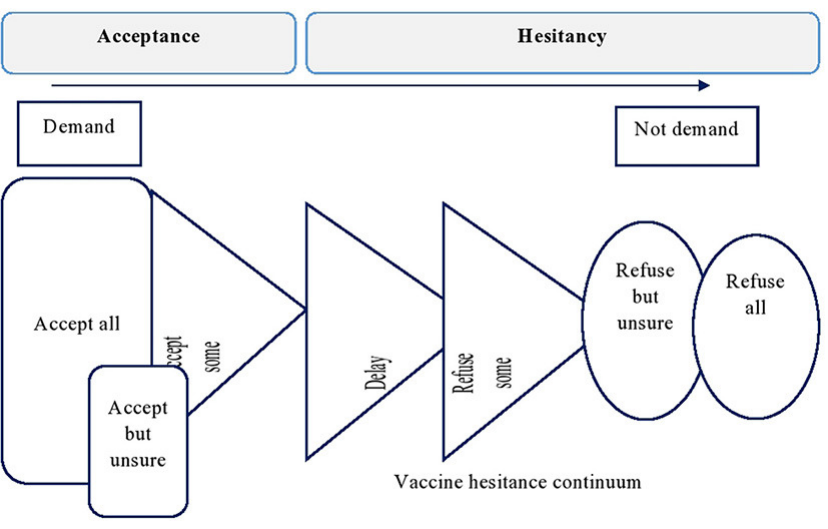
The continuum of vaccine hesitancy and acceptance of all vaccines. [Source (18)].

The term “vaccine hesitancy” refers to “delay in acceptance or refusal of vaccines despite the availability of vaccine services (6, 18, 19).” In this review, for articles that did not provide general acceptance of the vaccine among study participants, the prevalence of vaccine acceptance was calculated based on the response of the participants. The participant responded strongly agree, agree, completely agree, accept, all, accept, some accept, and yes to the questions were considered as accepted. Finally, the prevalence was calculated based on the frequency of responses and the total number of respondents. The same principle was applied to studies which reported results based on the Likert scale and others (18) (Figure 1).

### Statistical procedures and data analysis

The pooled acceptance rate of the COVID-19 vaccine was performed using Comprehensive Meta-Analysis (CMA) version 3.0 statistical software. Forest plots and random-effects models were used to determine and visualize the pooled acceptance rate of the COVID-19 vaccine. The Cochran Q-test (Q) and *I*-Squared test (*I*^2^ statistics) were used to evaluate the heterogeneity between the included articles. Then, heterogeneity was classified into low (*I*^2^ index <25%), medium (*I*^2^ index ranging from 25 to 75%), and high heterogeneity (*I*^2^ index > 75%). The random-effects model was used to analyze the data. Furthermore, subgroup analysis was performed based on the year of publication, survey period (when the study was conducted), and study area.

Sensitivity analysis was used to determine the differences in pooled effects by dropping studies that were found to influence the summary estimates, including extreme sample sizes and outcomes.

## Results

### Study selection

**Figure 2:**
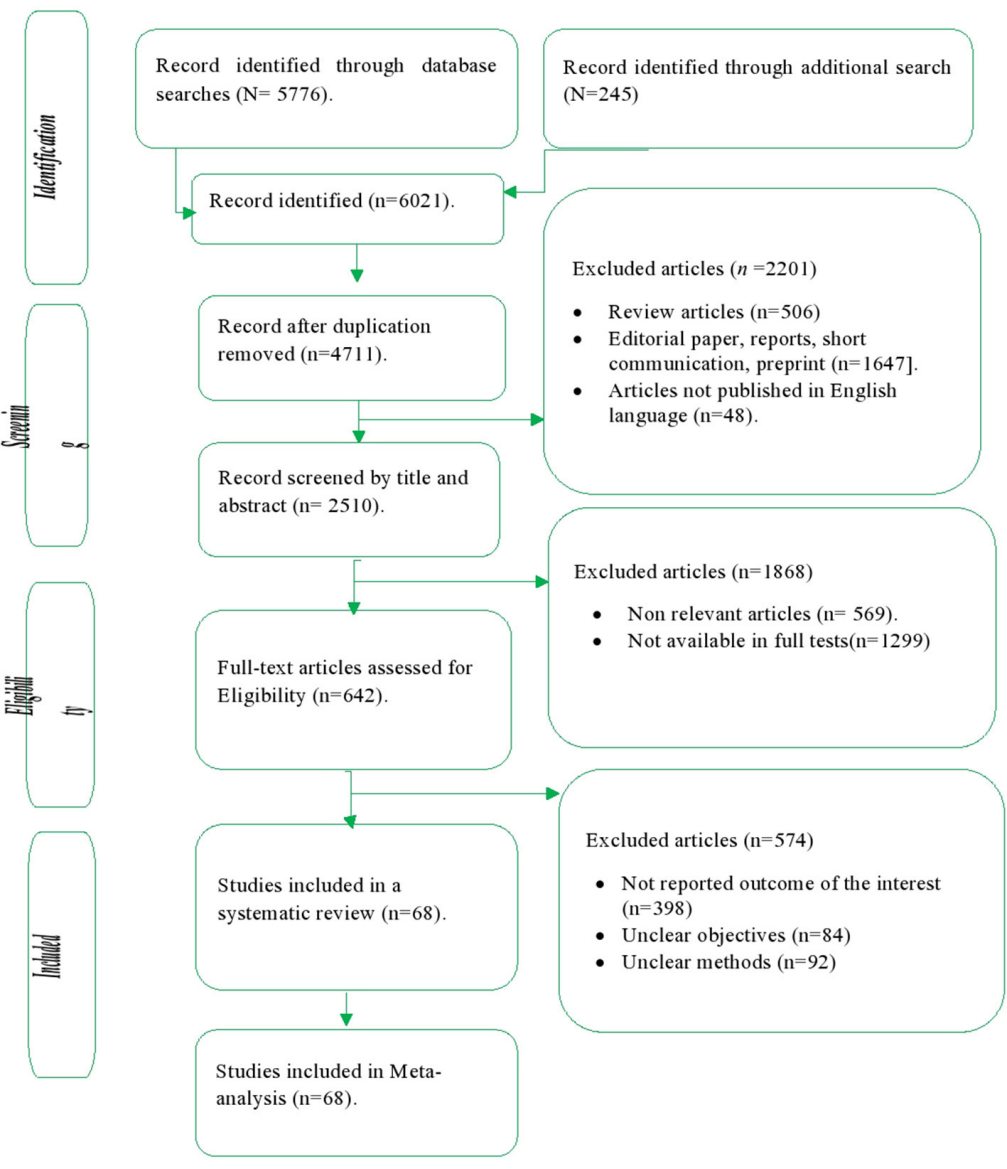
Study selection process of included articles for systematic review and meta analysis, 2021.

A total of 6,021 short communications, original articles and editorial articles were searched through electronic databases from PubMed, Scopus, Web of Science, Embase, CINAHL, and Google scholars. The articles were searched from February 01 to March 29, 2021 and May 02 to June 26, 2022 on PubMed, Scopus, Embase, and Google Scholars, while the search on Web of Science, CINAHL, and Google was made from 15 February to 31 March 2021. Then, 1,310 duplicate articles were excluded. Furthermore, 2201 articles were excluded after initial selection based on abstracts and titles. Furthermore, 599 articles were excluded after eligibility for full text articles (*n* = 601). Finally, a total of 68 articles were included in the systematic review and meta-analysis (Figure 2).

### Characteristics of the included articles

**Figure 3:**
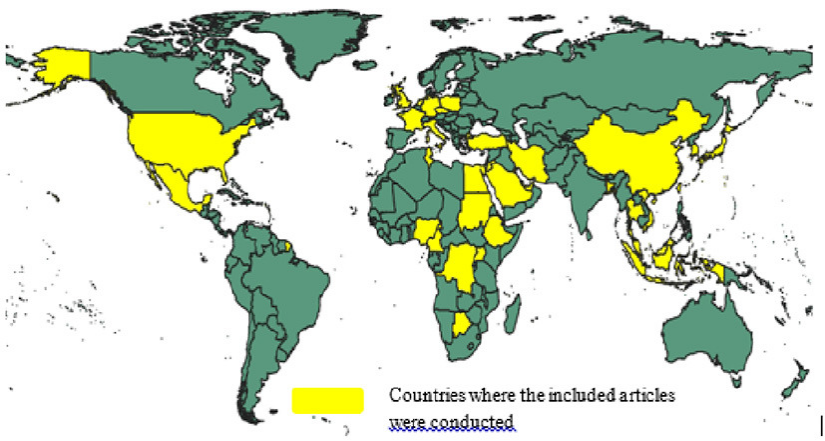
Countries of the world where the included articles were conducted.

Among the included articles, 35 (50%) had high quality, while the rest (50%) had moderate quality, based on the JBI critical appraisal tools for the prevalence study (17) (Supplementary file III). 143,111 study participants were included in 68 articles, which were published from 2020 to 2022. The included studies were conducted in 38 countries around the world (Figure 3).

Eight studies (14, 20–26) were conducted in China, six studies (27–32) in Saudi Arabia, four studies (2, 33–35) in United States, four studies (36–38) in United Kingdom, and four studies (39–42) in Turkey. Additionally, three studies were conducted in each Malaysia (43–45) and Kuwait (27, 46). Two studies conducted in each Qatar (47, 48), Italy (15, 49), Jordan (27, 50), Bangladesh (51, 52), Ethiopia (53, 54), Taiwan (55, 56), and Germany (57, 58).

However, only one study was conducted in each of the following countries; Republic of Congo (59), Japan (60), Poland (10), Cameroon (7), Israel (61), Mexico (62), Malta (63), Scotland (6), Indonesia (64), England (65), South Korea (66), Iran (67), Nigeria (68), Tunisia (69), Netherlands (70), Thailand (71), Vietnam (72), United Arab Emirates (73), Botswana (74), Sudan (75), Czechia (76), Uganda (77), France (78), and in Egypt (79).

The included studies were cross-sectional studies with a sample size ranging from 123 (63) to 23,582 (31) study participants. In general, the overall global acceptance rate of the COVID-19 vaccine, regardless of occupation, was 63.4% and ranged from 15.4% (7) to 95.6% (14) (Supplementary File IV).

### COVID-19 vaccine acceptance

This systematic review and meta-analysis was performed using Comprehensive Meta-Analysis (CMA) version 3 statistical software to determine pooled COVID-19 vaccine acceptance and hesitancy rates.

### The overall pooled prevalence/rate of COVID-19 vaccine acceptance

**Figure 4:**
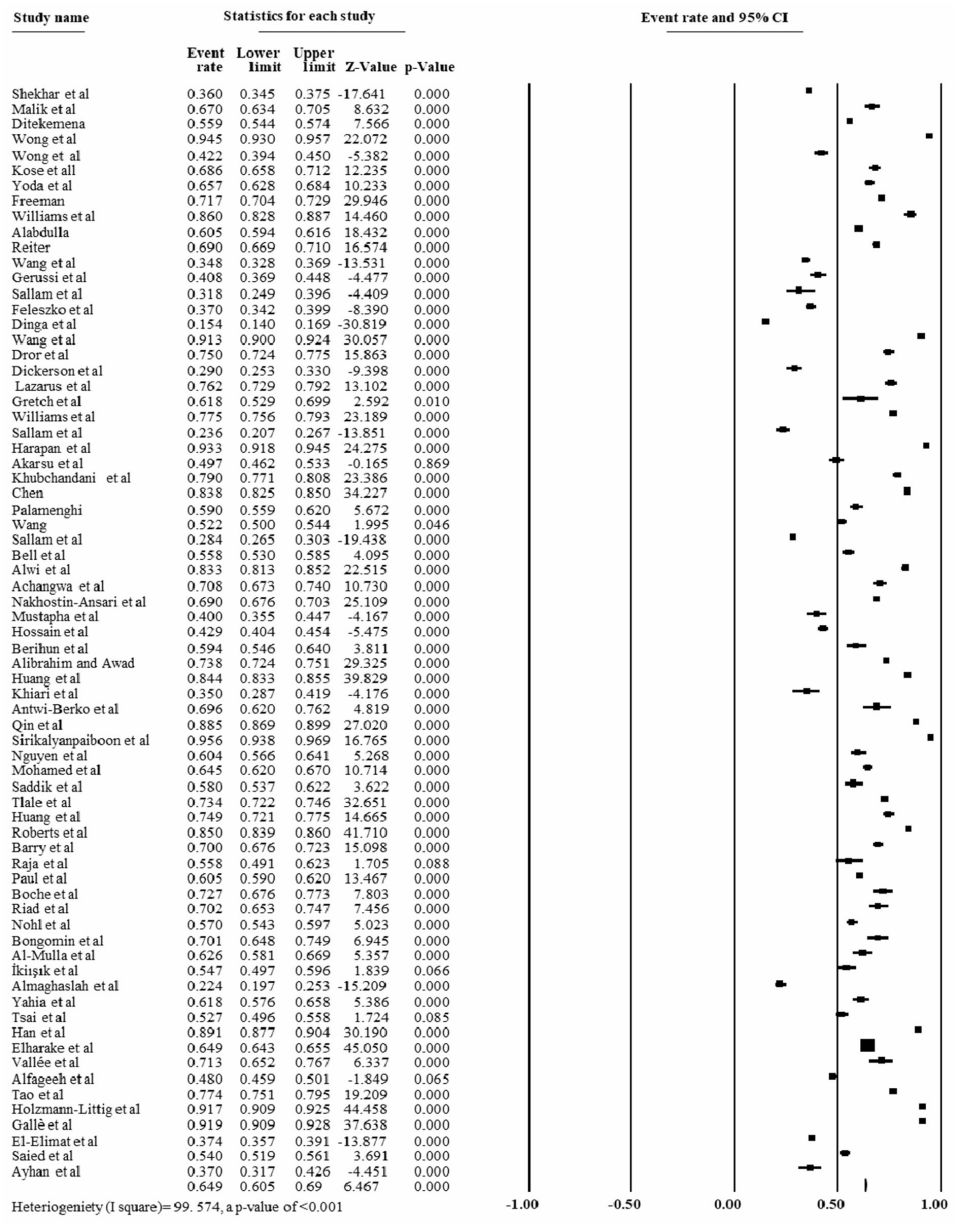
Forest plot shows the overall pooled COVID-19 vaccine acceptance rate, 2022.

The pooled prevalence of COVID-19 vaccine acceptance rate was found to be 64.9% [95% CI: 60.5 to 69.0%]; *I*^2^ = 99.57% with a *p*-value of < 0.001 (Figure 4).

### Subgroup analysis of the pooled prevalence of COVID-19 vaccine acceptance rate

**Figure 5:**
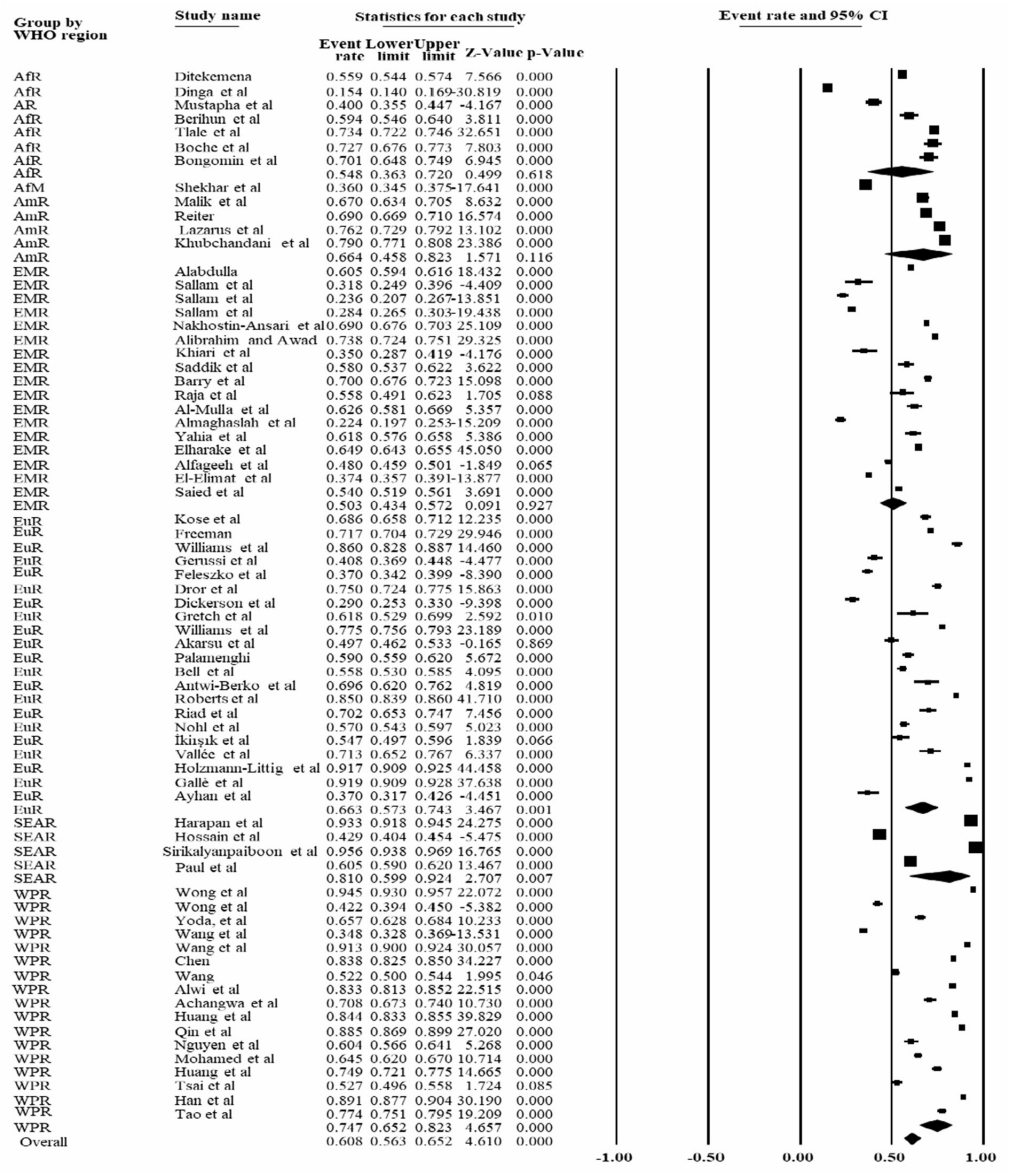
Forest plot shows the subgroup analysis of the pooled COVID-19 vaccine acceptance rate based on World Health Organization classification of the region 2022. ArR, African region; AmR, American region; EMR, Eastern Mediterranean Region; SEAR, South East Asian Region; WPR, Western Pacific Region; EuR, European Region.

Based on the subgroup analysis based on the World Health Organization's Region, the overall pooled prevalence of COVID-19 vaccine acceptance rate was 60.8% [95% CI: 56.3, 65.2%]. The lowest prevalence of COVID-19 vaccine acceptance rate was reported in the Eastern Mediterranean Region, accounting for 60.8% [95% CI: 43.4, 57.2%], whereas the highest prevalence was reported in the South East Asian Region, which accounted for 81.0% [95% CI: 59.9, 92.4%] (Figure 5).

**Figure 6:**
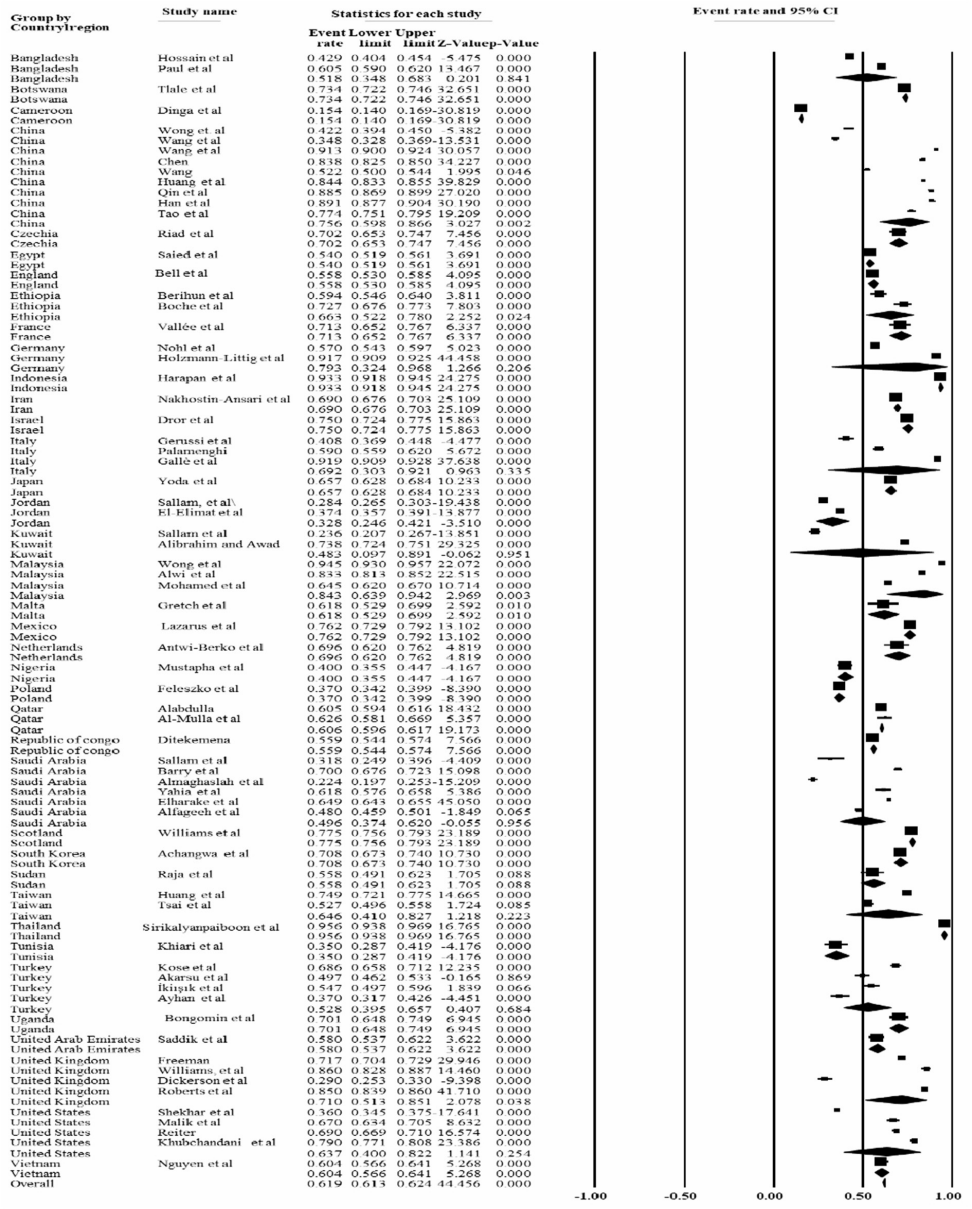
Forest plot shows the subgroup analysis of the pooled COVID-19 vaccine rate based on the country where the studies were conducted, 2022.

Based on the countries where the study was conducted, the lowest prevalence of COVID-19 vaccine acceptance rate was reported in Cameroon, accounted for 15.4% [95% CI: 14.0, 16.9], while the highest prevalence [95.6% (95% CI: 93.8, 96.9%] was reported in Thailand followed by Indonesia [93.3% (95% CI: 91.8, 94.5%] (Figure 6).

**Figure 7:**
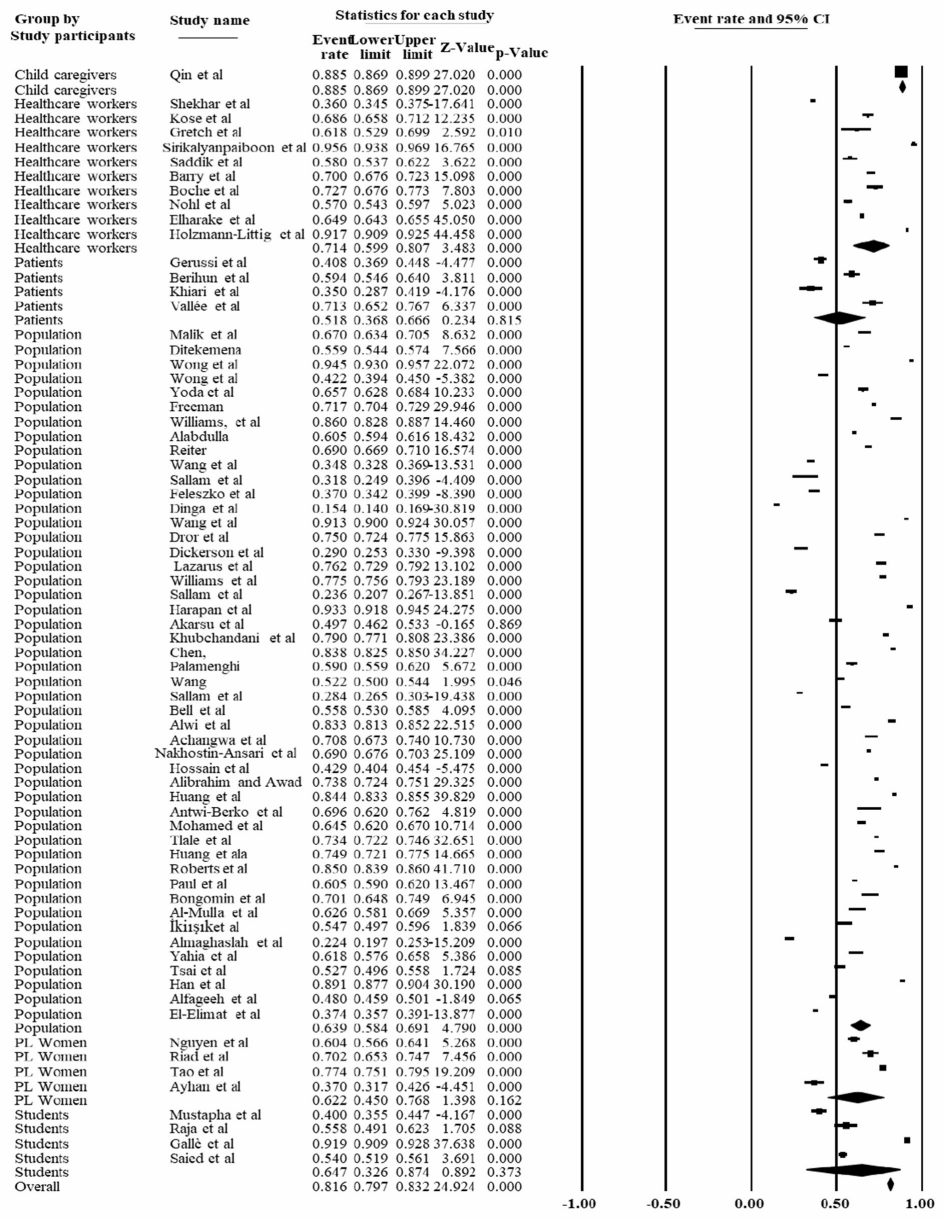
Forest plot shows the subgroup analysis of the pooled COVID-19 vaccine rate based on the study participants, 2022.

Based on the study participants, the highest COVID-19 vaccine acceptance rate was reported among healthcare workers, which accounted for 71.4% [95% CI: 59.9, 80.7%], followed by students accounted for 64.7% [95% CI: 32.6, 89.2%]. The lowest prevalence of COVID-19 vaccine acceptance rate was reported among patients [51.8% (95% CI: 36.8, 66.6%] (Figure 7).

**Figure 8:**
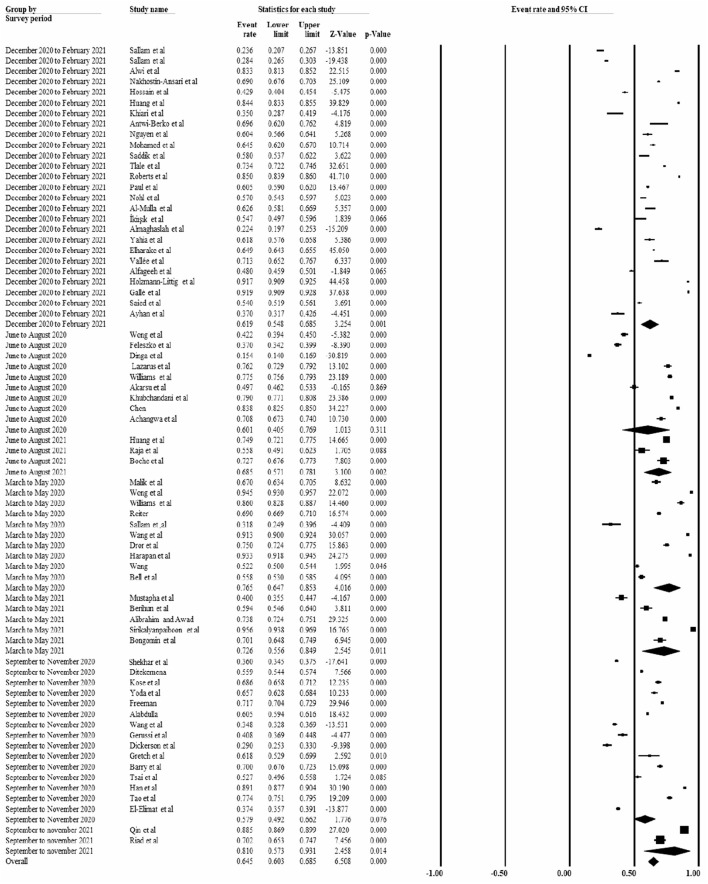
Shows the prevalence of the COVID-19 vaccine acceptance abased in the survey period, 2022.

Based on the survey period, the pooled prevalence of COVID-19 vaccine acceptance was 64.5% [95% CI: 60.3, 68.5%]. Relatively, the lowest prevalence [57.9% (95% CI: 49.2, 66.2%)] of vaccine acceptance was reported from September to November 2020, whereas the highest prevalence [81.0% (95% CI: 57.3, 93.1%] was reported between September to November 2021 (Figure 8).

### Sensitivity analysis

Sensitivity analysis was performed by removing low outcome, high outcome, and small sample sizes. However, the sensitivity analysis did not show a substantial change in the prevalence of COVID-19 acceptance compared to the pooled prevalence without sensitivity analysis [61.1% (95% CI 53.8 to 67.9%)] (Table 1).

**Table 1:** Results of sensitivity analysis for COVID-19 vaccine acceptance, 2022.

**Criteria**	**Acceptance rate/prevalence**	**Heterogeneity**	**95% Confidence interval**		**P-value**
			**Upper limit**	**Lower limit**	
After removing three articles with small sample size	65.2%		60.8	69.3	< 0.001
After removing one article with small sample size	64.85		60.0	69.3	< 0.001
After removing one article with low outcome	65.5%		61.5	69.4	< 0.001
After removing four articles with high prevalence rate	62.0%		57.8	66.1	< 0.001
After removing one article with low and four articles with high prevalence rate	65.8%		58.8	66.6	< 0.001

## Discussion

We conducted a systematic review and meta-analysis using data extracted from 68 studies conducted on 143,111 study participants. The study revealed that the pooled prevalence of COVID-19 vaccine acceptance was 64.9% [95% CI of 60.5 to 69.0%]. Some studies were conducted by the same authors across various countries (6, 27). The sensitivity analysis was employed to assess the cause of high heterogeneity and found no substantial difference in the prevalence of COVID-19 vaccine acceptance.

The utility of the vaccine to control COVID-19 pandemics depends on the acceptance of the vaccine (80, 81). Currently, vaccine hesitancy represents a serious threat to health. Similarly, the current study found that the global pooled prevalence of COVID-19 vaccine acceptance was 64.9% [95% CI of 60.5 to 69.0%], which was lower than the finding of the global survey, which reported about 71.5% of COVID-19 vaccine acceptance rate (62). The possible reason for the disparity in the prevalence estimate could be related to the variation in the study participants or the survey period. The former study was mainly conducted in a specific study period, whereas the present study's findings depend on the studies conducted during COVID-19 pandemic.

The lowest prevalence of COVID-19 vaccine acceptance rate was reported in Cameroon [15.4% (95% CI: 14.0, 16.9], while the highest prevalence [95.6% (95% CI: 93.8, 96.9%] was reported in Thailand, followed by Indonesia [93.3% (95% CI: 91.8, 94.5%]. The variation may be due to the difference in sources of information and types of study participants. Because, the study conducted in Thailand involved healthcare workers, whereas the study conducted in Cameroon involved the general population.

Furthermore, the current study found a slight difference in the pooled prevalence of COVID-19 vaccine acceptance rate among the studies conducted in the United States [60.4% (95% CI 56.6, 64.1%)], United Arab Emirates [58.0% (95% CI 53.7, 62.2%)], Taiwan [64.6% (95% CI 41.0, 82.7%)], and Qatar [60.6% (95% CI 59.6, 61.7%)].

Similarly, there was slight difference in the prevalence of COVID-19 acceptance rate among the studies conducted in the United Kingdom [71% (95% CI: 51.3, 85.1%)], South Korea [70.8% (95% CI: 67.3, 74.0%)], Netherland [69.6% (95% CI: 62.0,76.2%)], Italy [69.2% (95% CI: 30.3, 92.1%)], Iran [69.0% (95% CI: 67.6, 70.3%)], France [71.3% (95% CI: 65.2, 76.7%)] and Czechia [70.2% (95% CI: 65.3, 74.7%)]. However, in some countries there was a lower prevalence, such as Cameroon and Jordan, which reported 15.4 and 32%, respectively. In general, the variation in the estimate of the vaccine acceptance rate may be due to the difference in the information and sociodemographic characteristics of the study participants (Supplementary File V).

Based on World Health Organization Region, the overall COVID-19 vaccine acceptance rate was 60.8% [95% CI: 56.3, 65.2%] that was slightly lower than our findings without subgroup analysis. The lowest COVID-19 vaccine acceptance rate was reported in the Eastern Mediterranean Region accounted for 60.8% [95% CI: 43.4, 57.2%], followed by the Western Pacific [74.7% CI: 65.2, 82.3%] and American region (66.4%: CI: 59.4, 82.3%).

However, the highest prevalence was reported in South East Asian Region, which accounted for 81.0% [95% CI: 59.9, 92.4%]. The variation in vaccine acceptance rate may be related to the level of risk perception, study participants involved, and access to information (Supplementary File VI).

Based on the survey period, the COVID-19 acceptance rate was 76.5, 60.1, 57.9, 61.9, 72.6, 68.5, and 81.0% for the articles conducted from March to May 2020, June to August 2020, September to November 2020, December 2020 to February 20211, March to May 2021, June to August 2021 and September to November 2021, respectively. This indicates that there is a decline in COVID-19 vaccine acceptance rate from March to November 2020. The current study is supported by various studies (country or region-specific studies), which reported a decline in willingness to accept COVID-19 vaccine (6, 13, 14).

Similarly, this finding was in line with the findings of another study, which reported a decline in the acceptance rate of the COVID-19 vaccine from more than 70.0% in March to <50% in October (82). However, there was an increasing in COVID-19 vaccine acceptance rate from December 2020 to November 2021. It could be related to an increase in awareness, a change in risk perception, and the round of vaccines given across the world. The variation in the vaccine acceptance rate based on the survey period is indicated in the figure below (Supplementary File VII).

In general, the current study found that there was a declining in COVID-19 vaccine acceptance rate in 2020 and increasing in 2021. However, the overall COVID-19-vaccine acceptance rate was 64.9%. This indicates that there is a need to improve community awareness in order to increase COVID-19-vaccine acceptance rate. The authors recommend the need to take appropriate actions to manage the COVID-19 pandemic. Thus, local and international government should take appropriate action in collaboration with non-governmental organizations and community members to build trust in the community and to ensure adequate vaccination coverage. Furthermore, transparent and effective communications are essential to reduce misinformation and vaccination hesitancy, build trust, and ensure adequate vaccination coverage (8). Additionally, novel decision models for vaccine selection need to be developed.

### Implications of finding

The current study revealed that only about six out of ten study participants accepted the COVID-19 vaccine. This indicates that even if the COVID-19 vaccine is developed, the issue of accepting or taking the developed vaccine and managing the pandemic may be difficult. Not only for COVID-19, it must be used as input and considered to control other pandemics. These findings can be used as an input for concerned bodies, including health program planners, researchers, policymakers, and decision-makers, to take appropriate actions that can contribute to vaccine acceptance, ensure adequate vaccination coverage, and promote health.

### Limitations

There was an unequal distribution of the studies conducted across the world. Furthermore, the acceptance rates of the COVID-19 vaccine in many countries of the world were not included because of the lack of studies that met the eligibility criteria. Similarly, as a result of variation in the unit of measurement/statistical analysis employed for data analysis, we could not able to determine the factors associated with COVID-19 acceptance rate. Furthermore, cross-sectional studies were included and causal relationships between the acceptance rate of the COVID-19 vaccine and the determinant factors cannot be established.

## Conclusion

This review found a decline in the acceptance rate of the COVID-19 vaccine in 2020 and increasing acceptance in 2021. About 6 in 10 study participants accepted COVID-19 vaccine that needs critical attention to manage the COVID-19 pandemic. This finding indicated that even if the COVID-19 vaccine is developed, the issue of accepting or taking the developed vaccine and managing the pandemic will be difficult unless appropriate measures are taken when it is necessary. Furthermore, we recommend further studies, particularly on the determinants or factors that lead to hesitancy.

## Data availability statement

The original contributions presented in the study are included in the article/Supplementary material, further inquiries can be directed to the corresponding author/s.

## Author contributions

DM conceived the idea and had a major role in the review, extraction, analysis of the data, writing, drafting, and editing of the manuscript. YD has contributed to data extraction, analysis, and editing. All authors read and approved the final version of the manuscript to be published and agreed on all aspects of this work.
