# Supplementary material for: Global COVID-19 vaccine acceptance rate: Systematic review and meta-analysis
Source: Front Public Health. 2022 Dec 8;10:1044193. doi: 10.3389/fpubh.2022.1044193 (PMC9773145; doi:10.3389/fpubh.2022.1044193)
Supplement: Supplementary file 1 [file Data_Sheet_1.docx]

**Supplementary File I: Search Strategy**

The Embase search strategy was made as follows: **(1**) COVID 19 ' OR ‘COVID 19’/exp OR Coronavirus OR coronavirus/exp OR ‘2019 nCOV’ OR ‘2019 nCOV’/exp OR ‘severe acute respiratory syndrome coronavirus 2' / exp OR 'SARS-COV-2' OR 'SARS-COV 2/exp; **(2)** Vaccine OR *vaccine/exp* OR *Vaccination* OR *Vaccination/exp OR Immunization OR Immunization/exp; **(3)** acceptance OR acceptance /exp; OR ‘hesitance’ OR ‘hesitance/exp’ OR ‘refusal OR ‘refusal/exp’;**(4)** 1 AND 2 AND 3.

The PubMed search strategy was performed as follows: **(1)** 'Corona virus' [MeSH Terms] OR 'Corona virus' [All Fields] OR 'Corona virus' [All Fields] OR 'COVID-19' [All Fields] OR 'SARS-2' [All Fields] OR 'Severe acute respiratory syndrome corona virus 2' [All Fields] OR '2019 nCOV' [All Fields] OR 'SARS COV-2' [All Fields] OR 'Corona virus' [All Fields] AND; **(2)** 'Vaccines' [MeSH Terms] OR 'Vaccine' [All Fields] OR 'Vaccinations' [All Fields] OR 'Vaccines' [All Fields] OR 'Vaccines' [All Fields]; **(3)** “acceptance” [MeSH Terms] OR “hesitancy” [All Fields] OR “refusal” [All Fields] OR “accepted” [All Fields] OR “willingness to accept” [All Fields]; **(4)** 1 AND 2 AND 3.

The **Scopus and Web of Science search strategy** was made using a combination of keywords and Boolean functions: **(1)** (*COVID-19 * OR *Corona virus* OR *nCOV * OR *SARS COV-2*; **(2)** Vaccines *OR *Vaccination *OR *vaccinates*); **(3)** (*acceptance*OR *hesitancy* OR *refusal* OR accepted *OR *willingness to accept*; **(4)** 1 AND 2 AND 3) or 1 AND (2 OR 3)

The search strategy from **Google Scholars and Google** was done using keywords such as (Corona virus, nCOV, SARS, COV-2, COVID-19) AND **(**Vaccines OR Vaccination OR Vaccinates); **(3)** (acceptance OR hesitancy OR refusal OR accepted OR willingness to accept; **(4)** 1 AND 2 AND 3.
