# Supplementary material for: Global COVID-19 vaccine acceptance rate: Systematic review and meta-analysis
Source: Front Public Health. 2022 Dec 8;10:1044193. doi: 10.3389/fpubh.2022.1044193 (PMC9773145; doi:10.3389/fpubh.2022.1044193)
Supplement: Supplementary file 3 [file Data_Sheet_3.docx]

**Supplementary File III**

Table quality assessment results of included studies.

| **References** | **JBI appraisal tools for the prevalence studies (criteria)** | | | | | | | | | ***Grade*** | ***Quality Category*** |
| --- | --- | --- | --- | --- | --- | --- | --- | --- | --- | --- | --- |
|  | *1* | *2* | *3* | *4* | *5* | *6* | *7* | *8* | *9* |  |  |
| [2] | x | ✓ | ✓ | ✓ | ✓ | ✓ | ✓ | ✓ | ✓ | 8/9 | High |
| [34] | x | ✓ | ✓ | ✓ | ✓ | ✓ | ✓ | ✓ | ✓ | 8/9 | High |
| [60] | x | ✓ | ✓ | ✓ | ✓ | ✓ | ✓ | ✓ | x | 7/9 | Moderate |
| [44] | ✓ | ✓ | ✓ | ✓ | ✓ | ✓ | ✓ | ✓ | ✓ | 9/9 | High |
| [20] | x | ✓ | ✓ | ✓ | ✓ | ✓ | ✓ | ✓ | ✓ | 8/9 | High |
| [40] | x | ✓ | ✓ | ✓ | ✓ | ✓ | ✓ | ✓ | x | 7/9 | Moderate |
| [61] | x | ✓ | x | ✓ | ✓ | ✓ | ✓ | ✓ | ✓ | 7/9 | Moderate |
| [37] | x | ✓ | ✓ | ✓ | ✓ | ✓ | ✓ | ✓ | ✓ | 8/9 | High |
| [6] | x | x | ✓ | ✓ | ✓ | ✓ | ✓ | ✓ | ✓ | 7/9 | Moderate |
| [48] | x | ✓ | ✓ | ✓ | ✓ | ✓ | ✓ | ✓ | x | 7/9 | Moderate |
| [35] | x | ✓ | ✓ | ✓ | ✓ | ✓ | ✓ | ✓ | x | 7/9 | Moderate |
| [21] | x | ✓ | ✓ | ✓ | x | ✓ | ✓ | ✓ | ✓ | 7/9 | Moderate |
| [15] | x | ✓ | ✓ | ✓ | ✓ | ✓ | ✓ | ✓ | ✓ | 8/9 | High |
| [28] | x | ✓ | ✓ | ✓ | ✓ | ✓ | ✓ | ✓ | ✓ | 8/9 | High |
| [10] | x | ✓ | ✓ | ✓ | ✓ | ✓ | ✓ | ✓ | ✓ | 8/9 | High |
| [7] | x | ✓ | ✓ | ✓ | ✓ | ✓ | ✓ | ✓ | ✓ | 8/9 | High |
| [14] | x | ✓ | ✓ | ✓ | ✓ | ✓ | ✓ | ✓ | ✓ | 8/9 | High |
| [62] | x | ✓ | x | ✓ | ✓ | ✓ | ✓ | ✓ | ✓ | 7/9 | Moderate |
| [38] | x | ✓ | ✓ | ✓ | ✓ | ✓ | ✓ | ✓ | ✓ | 8/9 | High |
| [63] | x | ✓ | ✓ | ✓ | ✓ | ✓ | ✓ | ✓ | ✓ | 8/9 | High |
| [64] | x | ✓ | ✓ | ✓ | ✓ | ✓ | ✓ | ✓ | x | 7/9 | Moderate |
| [6] | x | x | ✓ | ✓ | ✓ | ✓ | ✓ | ✓ | ✓ | 7/9 | Moderate |
| [28] | x | ✓ | ✓ | ✓ | ✓ | ✓ | ✓ | ✓ | ✓ | 8/9 | High |
| [65] | x | ✓ | ✓ | ✓ | ✓ | ✓ | ✓ | ✓ | ✓ | 7/9 | Moderate |
| [41] | x | ✓ | ✓ | ✓ | ✓ | ✓ | ✓ | ✓ | ✓ | 8/9 | High |
| [36] | x | ✓ | ✓ | ✓ | ✓ | ✓ | ✓ | ✓ | ✓ | 8/9 | High |
| [22] | x | ✓ | ✓ | ✓ | ✓ | ✓ | ✓ | ✓ | ✓ | 8/9 | High |
| [23] | x | ✓ | ✓ | ✓ | ✓ | ✓ | ✓ | ✓ | x | 7/9 | Moderate |
| [81] | ✓ | x | ✓ | ✓ | ✓ | ✓ | ✓ | ✓ | ✓ | 8/9 | High |
| [66] | x | ✓ | ✓ | ✓ | ✓ | ✓ | ✓ | ✓ | ✓ | 8/9 | High |
| [45] | x | ✓ | ✓ | ✓ | ✓ | ✓ | ✓ | ✓ | x | 7/9 | Moderate |
| [67] | x | ✓ | x | ✓ | ✓ | ✓ | ✓ | ✓ | ✓ | 7/9 | Moderate |
| [68] | x | ✓ | ✓ | ✓ | ✓ | ✓ | ✓ | ✓ | ✓ | 8/9 | High |
| [69] | x | x | ✓ | ✓ | ✓ | ✓ | ✓ | ✓ | ✓ | 7/9 | Moderate |
| [52] | x | ✓ | ✓ | ✓ | ✓ | ✓ | ✓ | ✓ | x | 7/9 | Moderate |
| [54] | x | ✓ | ✓ | ✓ | ✓ | ✓ | ✓ | ✓ | x | 7/9 | Moderate |
| [47] | x | ✓ | ✓ | ✓ | x | ✓ | ✓ | ✓ | ✓ | 7/9 | Moderate |
| [24] | x | ✓ | ✓ | ✓ | ✓ | ✓ | ✓ | ✓ | ✓ | 8/9 | High |
| [70] | x | ✓ | ✓ | ✓ | ✓ | ✓ | ✓ | ✓ | ✓ | 8/9 | High |
| [71] | x | ✓ | ✓ | ✓ | ✓ | ✓ | ✓ | ✓ | x | 7/9 | Moderate |
| [25] | ✓ | x | ✓ | ✓ | ✓ | ✓ | ✓ | ✓ | ✓ | 8/9 | High |
| [72] | x | ✓ | ✓ | ✓ | ✓ | ✓ | ✓ | ✓ | ✓ | 8/9 | High |
| [73] | x | ✓ | ✓ | ✓ | ✓ | ✓ | ✓ | ✓ | x | 7/9 | Moderate |
| [46] | x | ✓ | x | ✓ | ✓ | ✓ | ✓ | ✓ | ✓ | 7/9 | Moderate |
| [74] | x | ✓ | ✓ | ✓ | ✓ | ✓ | ✓ | ✓ | ✓ | 8/9 | High |
| [75] | x | x | ✓ | ✓ | ✓ | ✓ | ✓ | ✓ | ✓ | 7/9 | Moderate |
| [56] | x | ✓ | ✓ | ✓ | ✓ | ✓ | ✓ | ✓ | ✓ | 8/9 | High |
| [39] | x | ✓ | ✓ | ✓ | ✓ | ✓ | ✓ | ✓ | ✓ | 8/9 | High |
| [29] | x | ✓ | ✓ | ✓ | ✓ | ✓ | ✓ | x | ✓ | 7/9 | Moderate |
| [76] | ✓ | x | ✓ | ✓ | ✓ | ✓ | ✓ | ✓ | ✓ | 8/9 | High |
| [53] | x | ✓ | ✓ | ✓ | ✓ | ✓ | ✓ | ✓ | ✓ | 8/9 | High |
| [55] | x | ✓ | ✓ | ✓ | ✓ | ✓ | ✓ | ✓ | x | 7/9 | Moderate |
| [77] | x | ✓ | x | ✓ | ✓ | ✓ | ✓ | ✓ | ✓ | 7/9 | Moderate |
| [58] | x | ✓ | ✓ | ✓ | ✓ | x | ✓ | ✓ | ✓ | 7/9 | Moderate |
| [78] | x | x | ✓ | ✓ | ✓ | ✓ | ✓ | ✓ | ✓ | 7/9 | Moderate |
| [49] | x | ✓ | ✓ | ✓ | ✓ | ✓ | ✓ | ✓ | x | 7/9 | Moderate |
| [42] | x | ✓ | ✓ | ✓ | ✓ | ✓ | ✓ | ✓ | x | 7/9 | Moderate |
| [30] | x | ✓ | ✓ | x | ✓ | ✓ | ✓ | ✓ | ✓ | 7/9 | Moderate |
| [31] | x | ✓ | ✓ | ✓ | ✓ | ✓ | ✓ | ✓ | ✓ | 8/9 | High |
| [57] | x | ✓ | ✓ | ✓ | ✓ | ✓ | ✓ | ✓ | ✓ | 8/9 | High |
| [26] | x | ✓ | ✓ | ✓ | ✓ | ✓ | ✓ | ✓ | ✓ | 8/9 | High |
| [32] | x | ✓ | ✓ | ✓ | ✓ | ✓ | ✓ | ✓ | ✓ | 8/9 | High |
| [79] | x | ✓ | ✓ | ✓ | ✓ | ✓ | ✓ | ✓ | x | 7/9 | Moderate |
| [33] | ✓ | x | ✓ | ✓ | ✓ | ✓ | ✓ | ✓ | ✓ | 8/9 | High |
| [27] | x | ✓ | ✓ | ✓ | ✓ | ✓ | ✓ | ✓ | ✓ | 8/9 | High |
| [59] | x | ✓ | ✓ | ✓ | ✓ | ✓ | ✓ | ✓ | x | 7/9 | Moderate |
| [50] | x | ✓ | x | ✓ | ✓ | ✓ | ✓ | ✓ | ✓ | 7/9 | Moderate |
| [51] | x | ✓ | ✓ | ✓ | ✓ | ✓ | ✓ | ✓ | ✓ | 8/9 | High |
| [80] | x | x | ✓ | ✓ | ✓ | ✓ | ✓ | ✓ | ✓ | 7/9 | Moderate |
| [43] | x | ✓ | ✓ | ✓ | ✓ | ✓ | ✓ | ✓ | x | 7/9 | Moderate |

***Keys****: 1= appropriate sampling frame; 2= proper sampling technique; 3=adequate sample size; 4=study subject and setting description; 5=sufficient data analysis; 6= use of valid methods for the identified conditions; 7= valid measurement for all participants; 8= using appropriate statistical analysis, and 9= adequate response rate.*
