# Supplementary material for: Global COVID-19 vaccine acceptance rate: Systematic review and meta-analysis
Source: Front Public Health. 2022 Dec 8;10:1044193. doi: 10.3389/fpubh.2022.1044193 (PMC9773145; doi:10.3389/fpubh.2022.1044193)
Supplement: Supplementary file 4 [file Data_Sheet_4.docx]

Supplementary File IV:

Table 1: Overall characteristics of the articles included in the systematic review and meta-analysis, 2022.

| *References* | *Survey period* | *Publication year* | *Sample size* | *Acceptance rate* | *Hesitance rate* | *Occupation* | *Country* | *WHO Region* |
| --- | --- | --- | --- | --- | --- | --- | --- | --- |
| [2] | October to November 2020 | 2021 | 4080 | 36.0 | 64.0 | Healthcare workers | United States | American Region |
| [33] | May 2020 | 2020 | 672 | 67.0 | 33.0 | Adult population | United States | American Region |
| [59] | August to September, 2020 | 2021 | 4131 | 55.9 | 44.1 | Adult population | Republic of Congo | African Region |
| [43] | April, 2020 | 2020 | 1159 | 94.3 | 5.7 | Adult population | Malaysia | Western Pacific Region |
| [20] | July to August, 2020 | 2021 | 1200 | 42.2 | 57.8 | Adult population | China | Western Pacific Region |
| [39] | September, 2020 | 2020 | 1138 | 68.6 | 31.4 | Healthcare workers | Turkey | European |
| [60] | September 2020 | 2021 | 1100 | 65.7 | 34.3 | Adult population | Japan | Western Pacific Region |
| [36] | September to October 2020 | 2020 | 5114 | 71.7 | 28.3 | Adults population | United Kingdom | European |
| [6] | April, 2020 | 2020 | 527 | 86.0 | 34.0 | Elder adults and patients | United Kingdom | European |
| [47] | October to November 2020 | 2021 | 7821 | 60.5 | 39.5 | Adult population | Qatar | Eastern Mediterranean Region |
| [34] | May 2020 | 2020 | 2006 | 69.0 | 31.0 | Adult population | United States | American Region |
| [21] | August to September 2020 | 2021 | 2047 | 34.8 | 65.2 | Adult population | China | Western Pacific Region |
| [15] | September to November 2020 | 2021 | 599 | 40.8 | 59.2 | Patients | Italy | European |
| [27] | December 2020 | 2021 | 154 | 31.8 | 68.2 | Adult population | Saudi Arabia | Eastern Mediterranean Region |
| [10] | June 2020 | 2021 | 1066 | 37.0 | 63.0 | Adult population | Poland | European |
| [7] | May to August 2020 | 2021 | 2512 | 15.4 | 84.6 | Adult population | Cameroon | African Region |
| [14] | March 2020 | 2020 | 2058 | 91.3 | 8.7 | Adult population | China | Western Pacific Region |
| [61] | March to April 2020 | 2020 | 1112 | 75.0 | 25.0 | Adult population | Israel | European |
| [37] | October to December 2020 | 2021 | 535 | 29.0 | 71.0 | Adult population | United Kingdom | European |
| [62] | June 2020 | 2021 | 699 | 76.2 | 23.8 | Adult population | Mexico | American Region |
| [63] | September 2020 | 2020 | 123 | 61.8 | 38.2 | Healthcare workers | Malta | European |
| [6] | August 2020 | 2021 | 2016 | 77.5 | 22.5 | High risk population | Scotland | European |
| [27] | December 2020 | 2021 | 771 | 23.6 | 76.4 | Adult population | Kuwait | Eastern Mediterranean Region |
| [64] | March to April 2020 | 2020 | 1359 | 93.3 | 6.7 | Adult population | Indonesia | South East Asian Region |
| [40] | June to July, 2020 | 2020 | 759 | 49.7 | 50.3 | Adult population | Turkey | European |
| [35] | June, 2020 | 20021 | 1878 | 79.0 | 22.0 | Adult population | United States | American Region |
| [22] | May to June 2020 | 2021 | 3195 | 83.8 | 16.2 | Adult population | China | Western Pacific Region |
| [23] | March 2020 | 2021 | 2058 | 52.2 | 47.8 | Adults population | China | Western Pacific Region |
| [80] | December 2020 | 2021 | 2173 | 28.4 | 71.6 | Adults population | Jordan | Eastern Mediterranean Region |
| [65] | April to May 2020 | 2021 | 1252 | 55.8 | 44.2 | Parents and guardians | England | European |
| [44] | December 23–29, 2020 | 2021 | 1411 | 83.3 | 16.7 | Adult population | Malaysia | Western Pacific Region |
| [66] | May to June 2021 | 2021 | 710 | 70.8 | 29.2 | Adult population | South Korea | Western Pacific Region |
| [67] | December 2020 to February 2021 | 2022 | 4604 | 69 | 31 | Adult population | Iran | Eastern Mediterranean Region |
| [68] | March to June 2021 | 2021 | 440 | 40 | 60 | Students | Nigeria | African Region |
| [51] | February 2021 | 2021 | 1497 | 42.9 | 46.2 | Adult population | Bangladesh | South East Asian Region |
| [53] | 2021 | 2021 | 416 | 59.4 | 40.6 | Patients | Ethiopia | African Region |
| [46] | March to April 2021 | 2021 | 4147 | 73.8 | 26.2 | Adult population | Kuwait | Eastern Mediterranean Region |
| [24] | January to March 2021 | 2022 | 4227 | 84.4 | 15.6 | HCW, Students, population, | China | Western Pacific Region |
| [69] | February 2021 | 2021 | 200 | 35 | 65 | patients | Tunisia | Eastern Mediterranean Region |
| [70] | January to April 2021 | 2022 | 160 | 69.6 | 30.4 | Minority Ethnic Groups | Netherlands | European |
| [24] | November 2021 | 2022 | 1,724 | 88.46 | 11.54 | child caregivers, Children | China | Western Pacific Region |
| [71] | March 2021 to April 2021 | 2021 | 705 | 95.6 | 4.4 | Healthcare workers | Thailand | South East Asian Region |
| [72] | January to February 2021 | 2021 | 651 | 60.4 | 39.6 | Pregnant Women | Vietnam | Western Pacific Region |
| [45] | December 2021 | 2021 | 1406 | 64.5 | 35.5 | Adult population | Malaysia | Western Pacific Region |
| [73] | November 2020 and January 2021 | 2022 | 517 | 58 | 42 | Healthcare workers | United Arab Emirates | Eastern Mediterranean Region |
| [74] | February 2021 | 2022 | 5300 | 73.4 | 26.6 | Adult population | Botswana | African Region |
| [55] | June to September 2021 | 2022 | 957 | 74.9 | 25.1 | elderly people | Taiwan | Western Pacific Region |
| [38] | December 2020 | 2021 | 4,535 | 85 | 15 | Adult population | United Kingdom | European |
| [28] | November 2020 | 2021 | 1512 | 70 | 30 | Healthcare workers | Saudi Arabia | Eastern Mediterranean Region |
| [75] | June to July 2021 | 2022 | 217 | 55.8 | 44.2 | Students | Sudan | Eastern Mediterranean Region |
| [52] | January to February 2021 | 2021 | 4175 | 60.5 | 39.5 | Adult population | Bangladesh | South East Asian Region |
| [54] | June to July 2021 | 2022 | 319 | 72.73 | 27.27 | Healthcare workers | Ethiopia | African Region |
| [76] | August to October 2021 | 2021 | 362 | 70.2 | 29.8 | Pregnant and Lactating Women | Czechia | European |
| [57] | December 2020 to January 2021 | 2021 | 1296 | 57 | 43 | Healthcare workers | Germany | European |
| [77] | March to April 2021 | 2021 | 317 | 70.1 | 29.9 | High-risk populations | Uganda | African Region |
| [48] | February 2021 | 2021 | 462 | 62.6 | 37.4 | Employees and students | Qatar | Eastern Mediterranean Region |
| [41] | December 2020 | 2021 | 384 | 54.7 | 45.3 | Adult population | Turkey | European |
| [29] | January to February 2021 | 2021 | 862 | 22.4 | 77.6 | Adult population | Saudi Arabia | Eastern Mediterranean Region |
| [30] | January and March 2021 | 2021 | 531 | 61.8 | 38.2 | Adult population | Saudi Arabia | Eastern Mediterranean Region |
| [56] | October 2020 | 2021 | 1020 | 52.7 | 47.3 | Adult population | Taiwan | Western Pacific Region |
| [25] | November 2020 | 2021 | 2126 | 89.1 | 10.9 | Adult population | China | Western Pacific Region |
| [31] | October to December 2020 | 2021 | 23,582 | 64.9 | 35.1 | Healthcare workers | Saudi Arabia | Eastern Mediterranean Region |
| [78] | January 2021 | 2021 | 237 | 71.3 | 28.7 | Patients | France | European |
| [32] | December 2020 | 2021 | 2137 | 48 | 52 | Adult population | Saudi Arabia | Eastern Mediterranean Region |
| [26] | November 2020 | 2021 | 1392 | 77.4 | 22.6 | Pregnant and Lactating Women | China | Western Pacific Region |
| [58] | February 2021 | 2021 | 4500 | 91.7 | 8.3 | Healthcare workers | Germany | European |
| [49] | February to April 2021 | 2021 | 3226 | 91.9 | 8.1 | Students | Italy | European |
| [50] | November 2020 | 2021 | 3,100 | 37.4 | 62.6 | Adult population | Jordan | Eastern Mediterranean Region |
| [79] | January 2021 | 2021 | 2133 | 54 | 46 | Students | Egypt | Eastern Mediterranean Region |
| [42] | January to February 202 | 2021 | 300 | 37 | 63 | Pregnant and Lactating Women | Turkey | European |

*Keys: HCWs: Healthcare Workers*
