# Supplementary figures and images for: Global COVID-19 vaccine acceptance rate: Systematic review and meta-analysis
Source: Front Public Health. 2022 Dec 8;10:1044193. doi: 10.3389/fpubh.2022.1044193 (PMC9773145; doi:10.3389/fpubh.2022.1044193)

**Supplementary File V**


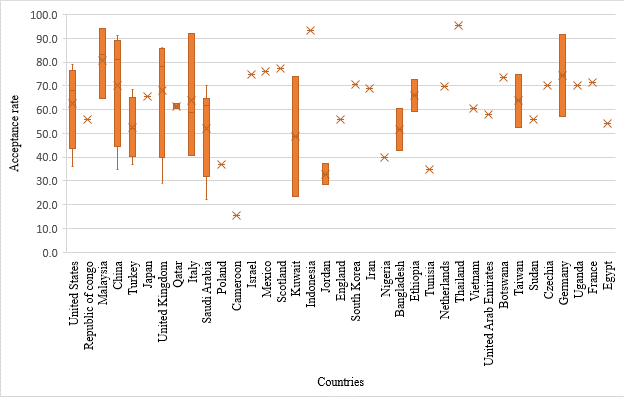


Figure 1: COVID-19: Vaccine acceptance rate among the included countries, 2022

Supplement: Supplementary file 5 [file Data_Sheet_5.docx]

**Supplementary File V**
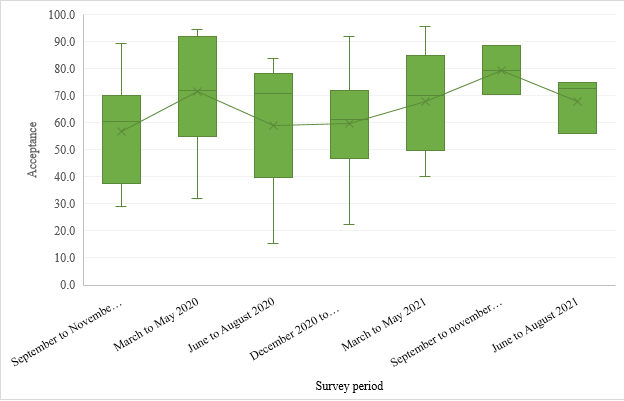
**II**

Figure 1: COVID-19 vaccine acceptance rate based on the survey period, 2022.

Supplement: Supplementary file 7 [file Data_Sheet_7.docx]
