# Supplementary material for: Global COVID-19 vaccine acceptance rate: Systematic review and meta-analysis
Source: Front Public Health. 2022 Dec 8;10:1044193. doi: 10.3389/fpubh.2022.1044193 (PMC9773145; doi:10.3389/fpubh.2022.1044193)
Supplement: Supplementary file 6 [file Data_Sheet_6.docx]

**Supplementary File VI.**


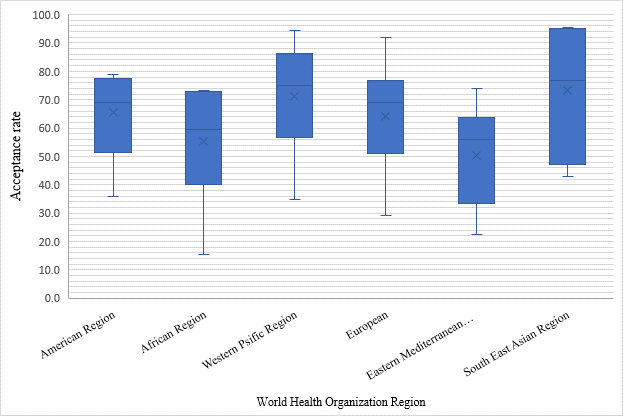


Figure 1: COVID-19 vaccine acceptance rate based on World Health Organization region of the world, 2022.
